# Socioeconomic position, treatment, and survival of non-Hodgkin lymphoma in Denmark – a nationwide study

**DOI:** 10.1038/bjc.2012.3

**Published:** 2012-02-07

**Authors:** B L Frederiksen, S O Dalton, M Osler, M Steding-Jessen, P de Nully Brown

**Affiliations:** 1 Research Centre for Prevention and Health, Glostrup University Hospital, DK-2600 Glostrup, Denmark; 2 Institute of Cancer Epidemiology, Danish Cancer Society, Strandboulevarden 49, DK-2100 Copenhagen Ø, Denmark; 3 Department of Haematology, Copenhagen University Hospital, DK-2100 Copenhagen Ø, Denmark

**Keywords:** non-Hodgkin lymphoma, socioeconomic position, survival, radiotherapy, chemotherapy, immunotherapy

## Abstract

**Background:**

Not all patients have benefited equally from the advances in non-Hodgkin lymphoma (NHL) survival. This study investigates several individual-level markers of socioeconomic position (SEP) in relation to NHL survival, and explores whether any social differences could be attributed to comorbidity, disease and prognostic factors, or the treatment given.

**Methods:**

This registry-based cohort study links clinical data on prognostic factors and treatment from the national Danish lymphoma database to individual socioeconomic information in Statistics Denmark including 6234 patients diagnosed with NHL in 2000–2008.

**Results:**

All-cause mortality was 40% higher in NHL patients with short *vs* higher education diagnosed in the period 2000–2004 (hazard ratio (HR)=1.40 (1.27–1.54)), and 63% higher in the period 2005–2008 (HR=1.63 (1.40–1.90)). Further, mortality was increased in unemployed and disability pensioners, those with low income, and singles. Clinical prognostic factors attenuated, but did not eliminate the association between education and mortality. Radiotherapy was less frequently given to those with a short education (odds ratio (OR)= 0.84 (0.77–0.92)), low income (OR=0.80 (0.70–0.91)), and less frequent to singles (OR=0.79 (0.64–0.96)). Patients living alone were less likely to receive all treatment modalities.

**Conclusion:**

Patients with low SEP have an elevated mortality rate after a NHL diagnosis, and more advanced disease at the time of diagnosis explained a part of this disparity. Thus, socioeconomic disparities in NHL survival might be reduced by improving early detection among patients of low SEP.

Over the past decades, survival rates for patients with non-Hodgkin lymphoma (NHL) have increased significantly in western countries (van de Schans *et al*, 2011a, 2011b). In Denmark, the 5-year age-standardised relative survival rate among males and females who were diagnosed between 1999 and 2003 was 53% and 58%, respectively, compared with rates of 28% and 32%, respectively, among patients who were diagnosed between 1974 and 1978 (Engholm *et al*, 2010). This improvement is mainly the result of advances in NHL treatment and better diagnostic tools facilitating earlier detection. Unfortunately, not all patients have benefited equally from the advances in NHL survival. A handful of studies have shown that persons with low socioeconomic position (SEP) experience poorer survival rates after a NHL diagnosis (Ewing *et al*, 2003; Bray *et al*, 2008; Rachet *et al*, 2008; Roswall *et al*, 2008; Wang *et al*, 2008; Kent *et al*, 2010). These disparities may be caused by several factors, including differences in the stage of lymphoma at the time of diagnosis (Frederiksen *et al*, 2010), comorbidity (Roswall *et al*, 2008), and the treatment given (Cronin *et al*, 2005). Elucidation of the relative roles of these factors could guide interventions to reduce disparities in survival.

Limitations in the previous evaluation of the role of SEP in NHL survival have been the dependence on area-based markers of SEP (Ewing *et al*, 2003; Bray *et al*, 2008; Rachet *et al*, 2008; Wang *et al*, 2008; Kent *et al*, 2010), or the lack of clinical data on prognostic factors and treatment (Rachet *et al*, 2008; Roswall *et al*, 2008).

Thus, in the present study, we investigate several individual-level markers of SEP in relation to NHL survival in a nationwide clinical database of nearly all NHL patients diagnosed in Denmark between 2000 and 2008, and examine whether any social differences could be attributed to comorbidity, disease and prognostic factors, or the treatment given.

## Materials and methods

### Study population

The study population was derived from the Danish national lymphoma database, LYFO, which includes more than 90% of patients diagnosed in Denmark with *de novo* NHL (Danish Lymphoma Group, 2010). The data are collected from questionnaires filled in by the medical doctors in all 13 haematological departments in Denmark, who diagnose and treat NHL. It is obligatory for all lymphoma-treating departments to report cases of lymphoma to the LYFO database, and validation of the data entry fields is performed instantly. In addition, a number of key data are validated against other central registries (Cancer registry and Pathology database) confirming date of diagnosis, histology, and stage. The LYFO database included 6596 persons born between 1920 and 1982, and diagnosed with NHL between 2000 and 2008. We excluded 63 patients who were under 25 years of age and therefore considered not to have established their final educational level and income. Further, a total of 362 persons (5%) for whom there were no achievable information on either highest attained education, cohabiting status, or disposable income 1 year before the diagnosis of lymphoma were excluded, leaving 6234 persons for analysis. Of these, 2670 (43%) were diagnosed with diffuse large-cell B-cell lymphoma and other high-grade B-cell subtypes, and 2490 (40%) with follicular and other indolent lymphomas.

### Exposure variables

The socioeconomic data were derived by linkage to the Central Population Registry and the population-based Integrated Database for Labour Market Research (IDA) in Statistics Denmark, by means of a unique personal 10-digit identifier, which is given to all persons residing in Denmark for more than 3 months (Baadsgaard and Quitzau, 2011; Jensen and Rasmussen, 2011; Petersson *et al*, 2011). Thus information on cohabitation status, education, and income were obtained for each patient, and affiliation to the work market was obtained for patients below 65 years of age (the typical age of retirement). Cohabitation status was categorised as living alone and living with partner. Education was categorised in three groups, as short education (i.e., mandatory education of up to 7 and 9 years for patients born before and after 1 January 1958, respectively), medium education (between 8/10 and 12 years – latest grades of primary school, secondary school, and vocational education), and higher education (over 12 years). Household income after taxation and interest per person, as defined in IDA, was adjusted for number of persons in the household and deflated according to the 2000 value of the Danish crown (DKK). Yearly variation in income was accounted for by calculating the average income in the 5 years before the diagnosis. Affiliation to the work market was categorised as working, not working (unemployed and disability pensioners), and anticipatory pension. Analyses of affiliation to the work market were restricted to those under the age of 65, as non-working was assumed for those above 65, the typical age of retirement.

### Outcome variables

The primary study endpoint was death from any cause. Patients were followed from date of diagnosis until death, emigration, or 31 December 2009, whichever came first. Dates of death and emigration were obtained from the Central Population Registry, as were age and sex (Pedersen, 2011). Additional endpoints included whether or not the patient received chemotherapy, radiotherapy, or immunotherapy (the latter only for patients diagnosed from 2005, where Rituximab was implemented routinely in the therapy of all subgroups of B-cell lymphoma).

### Other variables

A Charlson Comorbidity Index (CCI) was generated by linking the personal identification number to the files of the Danish National Patient Register (Charlson *et al*, 1987). Hereby full histories of diseases leading to hospitalisations and outpatient visits from 1978 to 1995, respectively, accumulated up to the year preceding the lymphoma diagnosis were obtained for each individual. The information in the Register includes dates of admission and discharge, and diagnoses coded according to the Danish-modified versions of the ICD-8 and, from 1994, ICD-10 (Lynge *et al*, 2011).

Clinical variables were provided through the LYFO database and included Ann Arbor stage, extranodal involvement, lactate dehydrogenase (LDH) level, ECOG performance status as scored by the physician at diagnosis, and IPI score, which is a prognostic composite measure generated from data on Ann Arbor stage, performance status, extranodal lesions, LDH level, and age (The International Non-Hodgkin's Lymphoma Prognostic Factors Project, 1993; Wilder *et al*, 2002). Further, histological subgroups were grouped according to aggressiveness and cell differentiation in diffuse large-cell B-cell lymphoma (DLBCL) and other high-grade B-cell subtypes, follicular lymphomas and other indolent lymphomas (LOW), T-cell lymphomas (PTCL), mantle cell lymphomas, and lymphomas of unknown subtype (NHLNOS).

### Statistical methods

Differences in the distribution of variables by level of education were analysed using the *χ*^2^ test. Cox proportional hazards regression models were used to compare mortality among socioeconomic groups using the PHREG procedure of SAS 9.1.3 (SAS Institute Inc., Cary, NC, USA). Inclusion of variables was performed as described in the tables. The cumulative hazard assumption was tested by Schoenfeld residuals and by testing the time-dependent coefficients (Therneau and Grambsch, 2000). Linearity of age was tested, and age was modelled as age and age^2^. Interactions of age, sex, and calendar period with education, cohabitation status, income, and attachment to work market were tested and are reported in the confounder-adjusted models when significant (Table 2). As the interaction between education and calendar period did not reveal any substantial difference regarding the structure of the relationship between the two factors, the interaction term was withdrawn from the analyses analysing mediation by clinical factors for a clearer presentation of the mediation (Table 3). Further, logistic regression models were used to examine the influence of the socioeconomic factors on 1-year mortality and treatment variables using the GENMOD procedure of SAS 9.1.3.

Possible clustering within hospital departments were accounted for using generalised estimating equations. A *P*-value of 0.05 was used as level of significance in all analyses.

## Results

### Socioeconomic position and survival from NHL

Table 1 shows descriptive and treatment characteristics of the 6234 NHL patients, overall and by level of education (Table 1). More men than women were diagnosed with NHL among persons with medium or higher education. The proportions of patients with low income, living alone, and no work market affiliation, and having high Ann Arbor stage, ECOG performance status, and IPI score were higher among persons with short education than for the groups of persons with medium or higher education. There were no substantial differences in extranodal involvement or level of LDH by educational group. In regard to treatment, more persons with higher education had radiotherapy, while no differences by educational groups were seen in chemotherapy and immunotherapy (Table 1).

**Figure 1:**
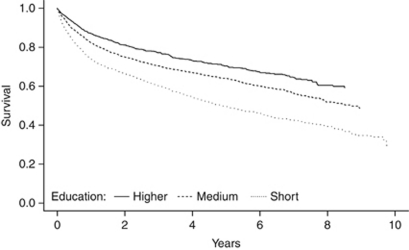
Kaplan–Meier survival curves (*N*=6234) by level of education.

Figure 1 displays the unadjusted Kaplan–Meier survival curve by level of education, and shows that the educational difference is mostly established during the first year after diagnosis. However, while the curves for medium and higher education are parallel hereafter, the gap in survival for those with short education seems to widen further. The odds ratio (OR) of dying within the first year since diagnosis was 1.71 (95% confidence interval (CI), 1.47–1.98) and 1.37 (95% CI, 1.22–1.53) in short and medium educated *vs* higher educated when controlling for age, sex, and year of diagnosis (data not shown).

The confounder-adjusted hazard ratio (HR) of all-cause mortality was 40% higher in NHL patients with short education diagnosed in the period 2000–2004 (HR, 1.40; 95% CI, 1.27–1.54) and 63% higher in the period 2005–2008 (HR, 1.63; 95% CI, 1.40–1.90) (Table 2). This increase in inequality with period was significant (*P*=0.009); however, interaction with period was restricted to the educational dimension of SEP, and was not found with regard to affiliation to the work market, income, or cohabiting status. There was an increased mortality in unemployed and disability pensioners and in those on anticipatory pension as compared with those working, and also in patients with low income, and singles as opposed to those living with a partner. Males living alone had significantly higher mortality than females living alone (*P*_interaction_=0.0008). Similar pattern of associations between SEP and all-cause mortality were obtained in the subsamples of the histological groups DLBCL and LOW; however, no interactions were found.

To explore whether the poorer survival experienced by patients with short education was mediated by higher comorbidity or more advanced disease at the time of diagnosis, these variables were included in the regression analyses in a dataset restricted to those patients with complete information hereof (*n*=5738) (Table 3). The confounder-adjusted HR of short *vs* higher education (HR, 1.48; 95% CI, 1.34–1.63) was only slightly affected by including the comorbidity variable (HR, 1.46; 95% CI, 1.32–1.61) (data not shown). Further, inclusion of performance status, Ann Arbor stage, extranodal involvement, and level of LDH decreased the HR to 1.30 (95% CI, 1.16–1.46), indicating some mediation. Both comorbidity and the clinical prognostic variables, except for extranodal involvement, were independently associated with survival. The mediation effect of including the composite measure of IPI instead of the individual prognostic factors was somewhat smaller (HR, 1.37; 95% CI, 1.24–1.50).

### Socioeconomic position and treatment

Differences in treatment may also mediate social differences in survival; however, this information on treatment was missing in up to 29% of patients in the dataset. Among patients without missing data, radiotherapy was less frequently given to those having a short *vs* long education (OR, 0.84; 95% CI, 0.77–0.92), income in the second (OR, 0.76; 95% CI, 0.68–0.85) or lowest quartile *vs* highest quartile (OR, 0.80; 95% CI, 0.70–0.91), and less frequent to singles *vs* those living with a partner (OR, 0.79; 95% CI, 0.64–0.96), when controlling for age, sex, year of diagnosis, comorbidity, clinical prognostic variables, NHL subtype, and department (Table 4). As some patient groups, namely those with the histological subgroups DLBCL and stage 1 or 2, and those with LOW and stage 1 or 2, were more frequently referred to radiotherapy; supplementary analyses were restricted to these groups. This exercise found similar trends in the point estimates; however, CIs were wider and thus did not reach significance in all cases (data not shown). Furthermore, singles were less frequently given all three treatment modalities, chemo-, radio-, and immunotherapy. No associations between education or income and receiving chemo- or immunotherapy were found. Including radiotherapy as a potential mediator in the survival analyses caused no changes in the association between education and all-cause mortality.

## Discussion

In this nationwide Danish study, we observed that survival rates among patients diagnosed with NHL were lower for those who had less education, low income, unemployed, and disability pensioners, in those on anticipatory pension, and in those living alone, and even lower for single males than for single females. These overall results also applied both to the NHL subgroups of the more aggressive lymphomas, DLBCL, and the less aggressive lymphomas, LOW. The educational differences in survival among NHL patients increased during the observation period. The lower survival rates among less-advantaged groups were explained in part by a higher likelihood of being diagnosed with advanced NHL, as expressed by several prognostic factors. Further, treatment with radiotherapy was less frequently given to those with short education, low income, and living single, and in general, singles were less likely to receive all forms of treatment. However, these treatment disparities did not seem to influence survival differences. The results suggest that socioeconomic disparities in NHL survival could be reduced by improving early detection among patients of low SEP.

One previous Danish study of NHL survival in relation to SEP was based on individual measures of SEP (Roswall *et al*, 2008). That study analysed socioeconomic differences in relative survival among NHL patients diagnosed between 1994 and 2003 comparing their mortality with the age-, sex-, and SEP-specific mortality of the total Danish population (Roswall *et al*, 2008). A relative survival in men and women with short education (basic or high school) of 48% and 58%, respectively, compared with a relative survival of 58% and 65%, respectively, in those with higher education was found. However, the study did not have access to clinical prognostic data or data on treatment, and thus was not able to explore the mechanisms of the social differences.

Social disparities in NHL survival were also found when based on area-based measures of SEP (Ewing *et al*, 2003; Bray *et al*, 2008; Rachet *et al*, 2008; Wang *et al*, 2008; Keegan *et al*, 2009; Kent *et al*, 2010). An US study on elderly NHL patients included clinical data on stage and lymph node site, as well as comorbidity and treatment with radio- or chemotherapy in the analyses. After adjustment for these factors, a gradient in survival by the composite socioeconomic status measure was seen favouring the better off (Wang *et al*, 2008). Furthermore, the lowest socioeconomic groups seemed less likely to receive radiotherapy, while chemotherapy was equally provided. These results are in line with our findings.

A Scottish investigation found that mortality was 19% and 10% higher in NHL patients from deprived and intermediate areas, respectively, compared with the most affluent areas, when adjusting for sex, age, and year of diagnosis (Bray *et al*, 2008). Prognostic information was only available for a very minor subsample of the study population in which no deprivation effect on survival was found, thus the mechanisms of social disparities could not be explored. Social disparities as measured by neighbourhood socioeconomic status also exist among Californian adolescents and young adults with NHL, even after adjustment for stage, nodality, chemotherapy, and radiotherapy (Kent *et al*, 2010).

The present study is one of the largest on SEP and survival after NHL. We used a model-building technique in which groups of variables were added sequentially in an attempt to distinguish which of these mechanisms were most relevant. Even though SEP was associated to comorbidity and comorbidity was associated to survival in our data, we found a rather modest mediating effect of this covariable. This modest effect may reflect that our measure of comorbidity was relatively crude. The CCI does not differentiate between the mildest and the most severe cases within the included categories of diseases, and in our case it was based on hospital discharge and outpatient visit diagnosis only, thus, patients with comorbidity treated in general practice only were registered with zero comorbidity.

We previously reported social disparities in the risk of being diagnosed with advanced disease (Frederiksen *et al*, 2010), which seemed to be consistent across NHL subtypes. Adjustment for the clinical prognostic variables reflecting advancement of disease at the time of diagnosis reduced the HRs, but a statistically significant association between education and NHL persisted. This is in accordance with some (Auvinen, 1992; Byers *et al*, 2008; Yu, 2009; Sprague *et al*, 2011) but not all (Hole and McArdle, 2002; Frederiksen *et al*, 2009) studies investigating the effect of stage on social disparities in survival from cancers. Further adjustments for radiotherapy did not affect the educational survival disparities in the subsample without missing data.

We investigated four different aspects of SEP: education, cohabitation, employment, and income, measuring different, but related aspects of socioeconomic stratification. This strengthens the interpretation of a true survival difference. We observed that the survival disparities as measured by education were growing larger during the observation period. This may be a spurious finding, as it is confined to the education variable only. Our finding that living single was associated with a reduced likelihood of receiving chemo-, radio-, and immunotherapy, after adjustment for education, age, sex, comorbidity, histology, and prognostic variables, is in accordance with studies on breast cancer and renal cancer showing less likelihood of getting the definite treatment in singles (Osborne *et al*, 2005; Hellenthal *et al*, 2009). It should be noted, however, that no significant difference in referral to receipt of chemotherapy by cohabitation status was seen, when only adjusting for age, sex, year of diagnosis, and education (the confounders). The difference was introduced by adjustment for the prognostic factors and histological subtype.

In the analyses of treatment, we excluded a substantial part of the patient group (29%) owing to missing information, which may lead to selection bias. However, the finding that high age, living single, and high comorbidity was associated with missing treatment information indicates that the strength of the associations observed between cohabitation and treatment might be underestimated. In a society like the Danish, with free and equal access to healthcare, personal economic barriers are not likely to explain this disparity. Rather, it may be that partners of NHL patients may encourage, support, and demand more active treatment for their relatives, and that physicians are more likely to provide the treatment to patients with these extra resources. However, it cannot be excluded that a higher proportion of patients living alone are regarded as more fragile as chemotherapy often requires care from your family. Treatment with immunotherapy has become standard in the treatment of most lymphomas. This treatment is expensive, and in the Danish health-care system, where reimbursement is not performed, each department has to pay from their budget and this might delay introduction of new expensive therapies in disfavour of vulnerable patient groups. Unfortunately, we were not able to explore social inequality in patients treated with immunotherapy before 2005 owing to the small numbers and missing information in this variable before 2005.

The strengths of this study include the availability of high-quality clinical information on a population basis, from a clinical database with a national coverage of more than 90% of NHL patients diagnosed in the period. Linkage with other administrative registries with information collected for purposes independent of the study hypotheses and with individual level data for the entire Danish population ensured minimal selection, information bias, and misclassification (Galobardes *et al*, 2006). Further, we measured SEP by several measures, thereby encompassing SEP from slightly different angles, and showing their different implications on health (Lahelma *et al*, 2004; Geyer *et al*, 2006).

Limitations include the exclusion of 362 patients with missing data on SEP variables (though these did not differ statistically from the study population with regard to age, sex, or survival (data not shown)), a potential risk that the ECOG performance status was scored in retrospect in a few number of patients, and a high number of missing data on treatment variables. We chose not to perform multiple imputation to replace these data, because we believe that the ‘missing at random’ assumption was not satisfied. Finally, we were unable to explore in detail whether disparities in the use of different chemotherapy regimes exist, as these data had even more missing information.

In summary, Danish patients with low SEP experienced an elevated mortality rate after a NHL diagnosis. More advanced disease at the time of diagnosis accounted for a moderate fraction of these disparities. While specific early detection programmes for NHL are currently not available in Denmark it seems reasonable to offer optimised diagnostic processes, securing early referral and navigation through different sectors of the health system to groups defined by low SEP or who live alone. Further, our findings that treatment with radiotherapy was less often provided to low SEP patients, and that patients living alone were less often given any lymphoma treatment should be further explored.

## Figures and Tables

**Table 1:** Baseline characteristics of 6234 persons born in 1920–1987 who were diagnosed with non-Hodgkin lymphoma in Denmark, 2000–2008, by education

	**Total**		**Short education, *N*=2081 (33%)**	**Medium education, *N*=2930 (47%)**	**Higher education, *N*=1223 (20%)**	
	***n***	**(%)**	**%**	**%**	**%**	***P***
*Sex (*n=*6234)*						
Men	3421	(55)	47	59	59	<0.0001
Women	2813	(45)	53	41	41	
						
*Age (years) (*n=*6234)*						
−49	785	(13)	5	15	19	<0.0001
50–59	1410	(23)	13	27	29	
60–69	1824	(29)	31	29	28	
70–79	1722	(28)	39	23	20	
80–	493	(8)	12	6	5	
						
*Cohabiting status (*n=*6234)*						
Living with partner	4383	(70)	64	73	75	<0.0001
Single	1851	(30)	36	27	25	
						
*Disposable income (*n=*6234)*						
Lowest	1509	(24)	41	20	7	<0.0001
Second quartile	1557	(25)	30	26	14	
Third quartile	1573	(25)	20	28	28	
Highest	1595	(26)	10	26	51	
						
*Affiliation to the work market (age<65 years,* n=*3136)*						
Working	2240	(71)	56	72	83	
Unemployed/disability pensioner	663	(21)	31	22	12	
Anticipatory pensioner	235	(8)	13	6	5	
						
*Charlson Comorbidity Index (*n=*6234)*						
None	4177	(67)	61	68	74	<0.0001
1	1026	(17)	19	16	13	
2	595	(10)	11	9	10	
⩾3	436	(7)	9	7	4	
						
*Year of diagnosis (*n=*6234)*						
2000–2004	3234	(52)	55	51	50	0.0014
2005–2008	3000	(48)	45	49	51	
						
*Ann Arbor stage (*n=*6051)*						
1	1425	(23)	22	23	25	0.0021
2	715	(12)	11	12	12	
3	1021	(16)	16	18	15	
4	2890	(46)	48	46	46	
						
*Two or more involved extranodal lesions (*n=*6234)*						
No	1076	(17)	19	17	17	0.2111
No	5158	(83)	81	83	83	
						
*Elevated level of LDH (*n=*5952)*						
Yes	2245	(38)	38	38	36	0.5926
No	3707	(62)	62	62	64	
						
*ECOG performance status (*n=*6169)*						
0	3248	(53)	45	54	63	<0.0001
1	1848	(30)	33	30	24	
2	539	(9)	12	8	5	
3	319	(5)	6	4	5	
4	215	(4)	5	3	3	
						
*IPI score (*n=*5738)*						
Low	2124	(37)	29	40	44	<.0001
Low–intermediate	1816	(32)	33	31	31	
High–intermediate	1129	(20)	22	19	17	
High	669	(12)	16	10	9	
						
*Histological subtype (*n=*6234)*						
DLBCL	2670	(43)	45	42	41	0.0005
LOW	2490	(40)	37	41	43	
MCL	341	(6)	6	5	5	
NHLNOS	319	(5)	6	5	4	
PTCL	414	(7)	6	7	7	
						
*Chemotherapy (*n=*5025)*						
Yes	4297	(86)	86	85	84	0.36
No	728	(14)	14	15	16	
						
*Radiotherapy (*n=*4987)*						
Yes	1479	(30)	27	29	36	<0.0001
No	3508	(70)	73	71	64	
						
*Immunotherapy (2005–2008,* n=*2187)*						
Yes	1111	(51)	47	53	50	0.11
No	1076	(49)	53	47	50	

Abbreviation: LDH=lactate dehydrogenase.

**Table 2:** Hazard ratios with corresponding 95% CIs for death due to all causes by four measures of socioeconomic position among 6234 patients diagnosed with non-Hodgkin lymphoma in Denmark, 2000–2008

	**NHL**	**DLBCL**	**LOW**
	***N*=6234**	***N*=2670**	***N*=2490**
	**HR (95% CI)**	**HR (95% CI)**	**HR (95% CI)**
*Education*			
	2000*–*2004	2000*–*2008	2000*–*2008
Short	1.40 (1.27*–*1.54)	1.37 (1.16*–*1.62)	1.52 (1.37*–*1.69)
Medium	1.12 (1.05*–*1.20)	1.27 (1.11*–*1.45)	1.18 (1.04*–*1.35)
Long	1	1	1
	2005*–*2008		
Short	1.63 (1.40*–*1.90)		
Medium	1.50 (1.30*–*1.74)		
Long	1		
			
*Cohabiting status*			
	Males	Both sexes	Both sexes
Living with partner	1	1	1
Single	1.42 (1.29*–*1.56)	1.26 (1.12*–*1.41)	1.36 (1.17*–*1.57)
	Females		
Living with partner	1		
Single	1.17 (1.08*–*1.26)		
			
*Affiliation to work market, age <65 years*			
Working	1	1	1
Unemployed/disability pensioners	1.68 (1.43*–*1.97)	1.81 (1.53*–*2.14)	1.52 (1.10*–*2.10)
Anticipatory pensioners	1.35 (1.06*–*1.73)	1.62 (1.10*–*2.39)	1.21 (0.75*–*1.95)
			
*Disposable income*			
First quartile	1.28 (1.12*–*1.46)	1.11 (0.95*–*1.31)	1.71 (1.27*–*2.30)
Second quartile	1.36 (1.16*–*1.60)	1.15 (0.94*–*1.42)	2.02 (1.51*–*2.70)
Third quartile	1.15 (1.02*–*1.29)	1.04 (0.92*–*1.17)	1.29 (0.96*–*1.75)
Fourth quartile	1	1	1

Abbreviations: NHL=non-Hodgkin lymphoma, DLBCL=diffuse large-cell B-cell lymphoma and other high-grade B-cell subtypes, LOW=follicular lymphomas and other indolent lymphomas, HR=hazard ratio, CI=confidence interval.

All analyses are adjusted for age, sex, year of operation, and for clustering at the department level. Cohabiting status is also adjusted for education; affiliation to work market and income are also adjusted for education and cohabiting status. The analyses of affiliation to work market include only patients aged <65 years at diagnosis (*N*=3136 NHL patients; *N*=1308 DLBCL patients; and *N*=1323 LOW patients).

**Table 3:** Hazard ratios with corresponding 95% CIs for death due to all causes by education and clinical variables among 6234 patients diagnosed with non-Hodgkin lymphoma in Denmark, 2000–2008

	**Adjusted for age, sex, and year**	**Multivariate (1)**	**Multivariate (2)**
***N*=5738**	**HR (95% CI)**	**HR (95% CI)**	**HR (95% CI)**
*Education*			
Short	1.48 (1.34–1.63)	1.30 (1.16–1.46)	1.37 (1.24–1.50)
Medium	1.28 (1.19–1.38)	1.20 (1.08–1.33)	1.22 (1.13–1.32)
Long	1	1	1
			
*Charlson Comorbidity Index*			
None	1	1	1
1	1.27 (1.17–1.38)	1.21 (1.09–1.33)	1.27 (1.18–1.37)
⩾2	1.60 (1.44–1.77)	1.53 (1.35–1.73)	1.60 (1.45–1.75)
			
*WHO performance status*			
0	1	1	
1	2.16 (2.02–2.30)	1.80 (1.65–1.96)	
2	4.34 (3.74–5.05)	3.31 (2.91–3.76)	
3	5.69 (4.67–6.95)	4.23 (3.53–5.07)	
4	9.40 (7.26–12.16)	7.37 (5.79–9.39)	
			
*Ann Arbor stage*			
1	1	1	
2	1.39 (1.07–1.79)	1.10 (0.81–1.50)	
3	1.65 (1.37–2.00)	1.18 (0.93–1.49)	
4	1.94 (1.75–2.15)	1.35 (1.18–1.53)	
			
*Two or more involved extranodal lesions*			
Yes	2.33 (2.14–2.53)	1.09 (0.98–1.21)	
No	1	1	
			
*Elevated level of LDH*			
Yes	1.79 (1.64–1.95)	1.71 (1.55–1.90)	
No	1	1	
			
*IPI score*			
Low (0–1)	1		1
Low–intermediate (2)	2.03 (1.80–2.28)		2.03 (1.80–2.30)
High–intermediate (3)	2.96 (2.67–3.28)		2.94 (2.64–3.28)
High (4–5)	6.23 (5.01–7.76)		6.17 (5.02–7.59)

Abbreviations: CI=confidence interval, HR=hazard ratio, LDH=lactate dehydrogenase.

All analyses are adjusted for age, sex, and year of diagnosis, and for clustering at the department level. The multivariate models are further adjusted for the other variables in the column. Multivariate model (1) without components of IPI combined and (2) with IPI.

**Table 4:** Adjusted odds ratios with corresponding 95% CIs of receiving chemo-, radio-, and immunotherapy among patients diagnosed with non-Hodgkin lymphoma in Denmark, 2000–2008

	**Chemotherapy**	**Radiotherapy**	**Immunotherapy, 2005–2008**
	**(4048/4698)**	**(1393/4662)**	**(1492/2134)**
**(Cases/*N*)**	**OR (95% CI)**	**OR (95% CI)**	**OR (95% CI)**
*Education*			
Short	1.09 (0.81–1.47)	0.84 (0.77–0.92)	0.92 (0.62–1.37)
Medium	0.99 (0.75–1.30)	0.82 (0.72–0.93)	1.12 (0.85–1.48)
Long	1	1	1
			
*Cohabiting status*			
Living with partner	1	1	1
Single	0.79 (0.65–0.97)	0.79 (0.64–0.96)	0.82 (0.70–0.96)
			
*Disposable income*			
First quartile	0.99 (0.72–1.36)	0.80 (0.70–0.91)	0.92 (0.65–1.30)
Second quartile	0.96 (0.74–1.25)	0.76 (0.68–0.85)	0.80 (0.63–1.01)
Third quartile	1.06 (0.81–1.39)	0.97 (0.75–1.24)	0.96 (0.73–1.27)
Fourth quartile	1	1	1

Abbreviations: OR=odds ratio; CI=confidence interval.

Cohabiting status is adjusted for education; income is adjusted for education and cohabiting status. In addition, all analyses are adjusted for age, sex, year of diagnosis, comorbidity, Ann Arbor stage, lactate dehydrogenase, ECOG performance status, extranodal involvement, and for clustering at the department level.
